# All Roads Lead to Susceptibility: The Many Modes of Action of Fungal and Oomycete Intracellular Effectors

**DOI:** 10.1016/j.xplc.2020.100050

**Published:** 2020-04-24

**Authors:** Qin He, Hazel McLellan, Petra C. Boevink, Paul R.J. Birch

**Affiliations:** 1 Key Laboratory of Horticultural Plant Biology (HZAU), Ministry of Education, Key Laboratory of Potato Biology and Biotechnology (HZAU), Ministry of Agriculture and Rural Affairs, Huazhong Agricultural University, Wuhan, Hubei 430070, China; 2 Division of Plant Sciences, School of Life Sciences, University of Dundee (at JHI), Invergowrie, Dundee DD2 5DA, UK; 3 Cell and Molecular Sciences, James Hutton Institute, Invergowrie, Dundee DD2 5DA, UK

**Keywords:** effectors, fungi, oomycete, immunity, defense, susceptibility factor

## Abstract

The ability to secrete effector proteins that can enter plant cells and manipulate host processes is a key determinant of what makes a successful plant pathogen. Here, we review intracellular effectors from filamentous (fungal and oomycete) phytopathogens and the host proteins and processes that are targeted to promote disease. We cover contrasting virulence strategies and effector modes of action. Filamentous pathogen effectors alter the fates of host proteins that they target, changing their stability, their activity, their location, and the protein partners with which they interact. Some effectors inhibit target activity, whereas others enhance or utilize it, and some target multiple host proteins. We discuss the emerging topic of effectors that target negative regulators of immunity or other plant proteins with activities that support susceptibility. We also highlight the commonly targeted host proteins that are manipulated by effectors from multiple pathogens, including those representing different kingdoms of life.

## Teaser

Recent years have seen increased efforts to identify the targets of intracellular effectors produced by fungi and oomycetes. This review describes the roles played by effector targets in the control of plant immunity and the associated modes of action by which fungal and oomycete effectors manipulate these regulatory processes in the host.

## Introduction

To successfully colonize plants, pathogenic microbes must suppress or evade different layers of immunity. Plant pattern recognition receptors (PRRs) recognize conserved microbe-associated molecular patterns (MAMPs) such as chitin and β-glucans. This leads to the amplification of defense responses designed to prevent pathogen growth and is termed pattern-triggered immunity (PTI). To combat PTI, pathogens secrete proteins called effectors, which may act either inside or outside plant cells, interacting with various host targets to block PTI and promote colonization. However, plants also possess Nod-like intracellular receptors (NLRs), which can detect the presence of certain effectors either through direct interaction or by monitoring changes in targeted host proteins. This recognition triggers an enhanced defense response. Thus, the precise effector complement of a pathogen is vital in determining the outcome of a host–pathogen interaction (Jones and Dangl, 2006).

Gram-negative bacterial plant pathogens have small effector sets of approximately 30 or more proteins that can be delivered into host cells by mechanisms such as the type III secretion system (Cunnac et al., 2009, Studholme et al., 2009). These effectors and their host manipulations have been well studied (Deslandes and Rivas, 2012, Feng and Zhou, 2012, Büttner, 2016, Macho, 2016, Khan et al., 2018, Lee et al., 2019) and thus are not the focus of this review, nor are the emerging areas of effectors delivered by insect (Rodriguez et al., 2017) or nematode (Lilley et al., 2018) pests. This review focuses on filamentous (fungal and oomycete) plant pathogens, which are thought to produce relatively large effector complements based on bioinformatic and transcriptomic analyses of their secretomes (Thordal-Christensen et al., 2018). A breakthrough for oomycete pathogens was the identification of the conserved amino acid motifs RxLR and LFLAK (Rehmany et al., 2005, Haas et al., 2009). These motifs define sets of several hundred intracellular effectors and have led to an upsurge in research on effector–host target interactions. For fungal plant pathogens, there are no such universal motifs, so the identification of bona fide intracellular effectors is a labor-intensive process initiated by the broader bioinformatic prediction of secreted proteins. Here, we focus on intracellular effector–host target interactions associated with filamentous phytopathogens. We review the current state of knowledge on the types of proteins and processes manipulated by effectors, and the modes of action of effectors. We also highlight increasing evidence for the targeting of so-called susceptibility (S) factors and of common proteins targeted by distantly related pathogen species.

## Which Proteins and Processes Do Effectors Target?

To determine which proteins and processes are targeted by filamentous pathogens, we collated data from the literature describing verified targets of intracellular effectors from fungi and oomycetes (Supplemental Table 1). These data reveal the host targets of 71 effector proteins, 41 of which are from oomycetes and 30 from fungi. These targets are classified according to their biological functions. Most of the targets are, or include, host proteins. However, three of the effectors target DNA. For example, the *Phytophthora sojae* effector CRN108 is reported to prevent heat-shock element (HSE)-mediated gene expression by binding to HSEs in gene promoters (Song et al., 2015).

**Figure 1:**
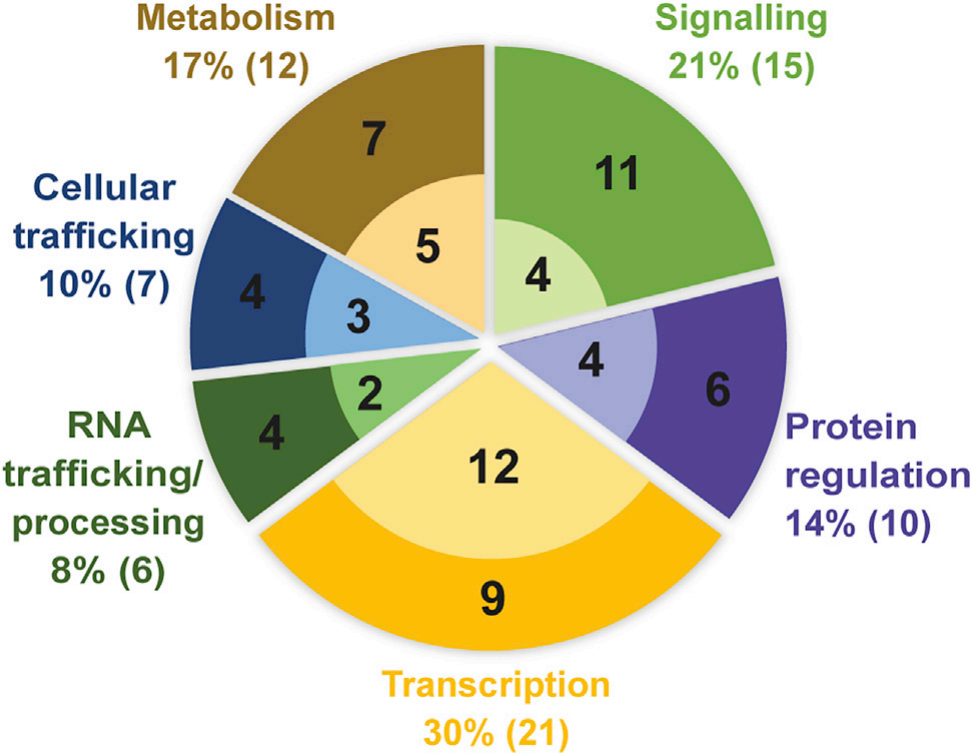
Processes Targeted by Filamentous Phytopathogen Effector Proteins. The pie chart shows the percentage of effectors that interact with host proteins from each biological category; total numbers of effectors are indicated in brackets. Within each pie segment, the numbers indicate oomycete (outer ring) or fungal (inner ring) effectors within that category.

Approximately 50% of the host protein targets of effector proteins are involved in transcription and signaling (Figure 1). This is perhaps unsurprising, as these functions are likely to be important for the regulation of plant immunity. The other 50% of effector targets are proteins involved in metabolism, cellular trafficking, protein regulation, or RNA trafficking/processing (Figure 1), all known to function in defense. Both fungi and oomycetes express effectors that target host proteins from each category. Fungal effector targets are enriched for involvement in transcription, whereas oomycete effector targets are enriched for roles in signaling (Figure 1). A key question is: how do effectors from filamentous phytopathogens manipulate these diverse processes?

## Effector Modes of Action

### Effectors with Enzyme Activities

**Figure 2:**
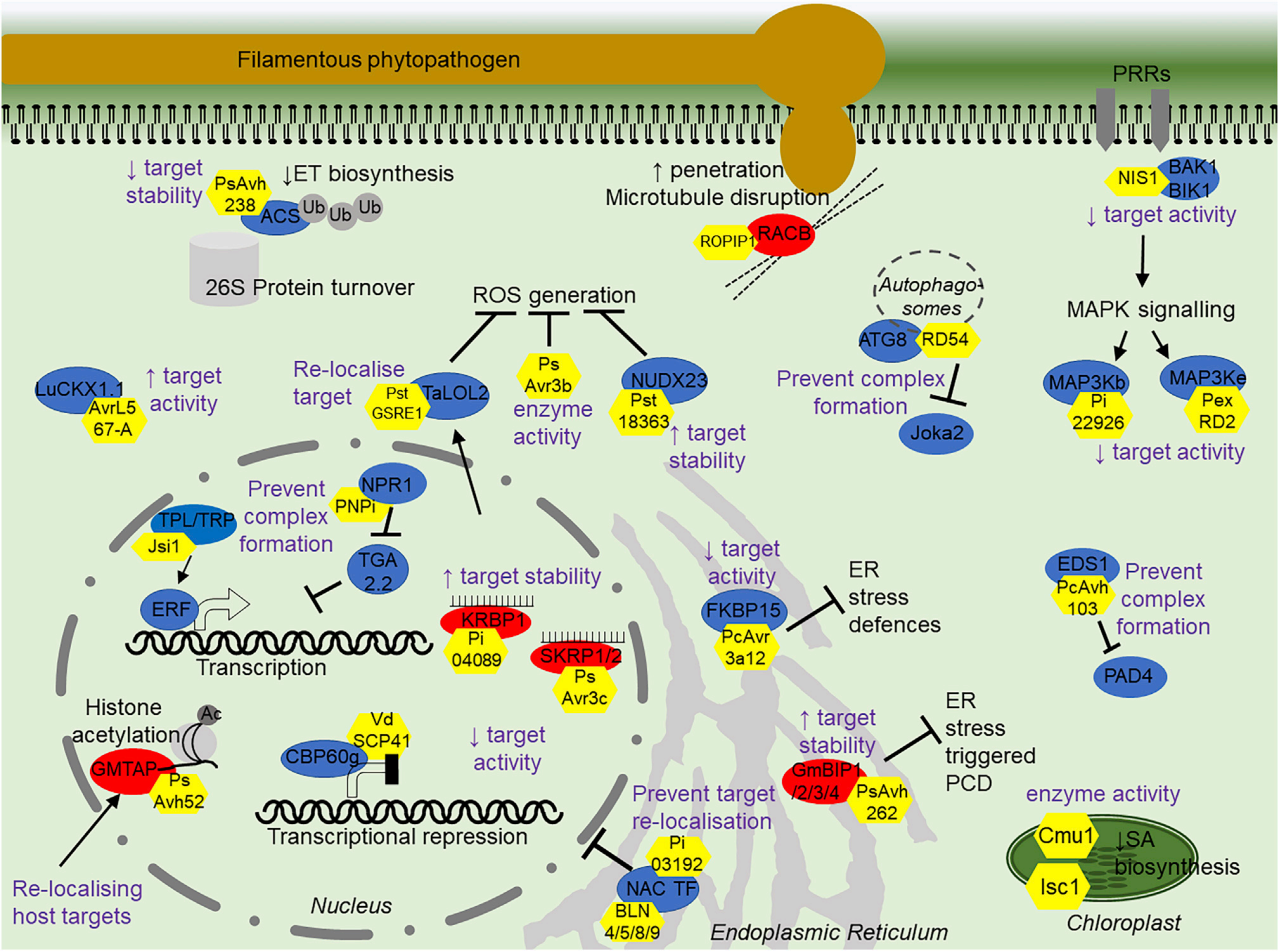
Plant Targets and Modes of Action of Filamentous Phytopathogen Effectors. Effectors are shown in yellow. Positive regulators of immunity are shown in blue, and negative regulators of immunity are shown in red. The mode of action of the effectors is written in purple text, with an upward arrow denoting increase and a downward arrow denoting decrease.

Many bacterial effectors are large proteins that contain regions with enzyme activities such as kinase or protease domains (Galán, 2009, Dean, 2011) and can directly modify host proteins accordingly. Only a handful of filamentous phytopathogen intracellular effectors possess known enzyme activities. These include a chorismate mutase (Cmu1) from *Ustilago maydis* (Djamei et al., 2011, Lanver et al., 2017) and isochorismatases (Isc) from the fungus *Verticillium dahlia* (VdIsc1) and the oomycete *P. sojae* (PsIsc1) (Liu et al., 2014) (Figure 2). These act inside plant cells and use different strategies to reduce the accumulation of the defense hormone salicylic acid (SA). Cmu1 redirects the pool of chorismate through the shikimate pathway to produce tyrosine and phenylalanine (Djamei et al., 2011), whereas Isc1 hydrolyzes the SA precursor isochorismate (Liu et al., 2014). The legume root oomycete pathogen *Aphanomyces euteiches* secretes the effector AeCRN13, which contains an HNH-like endonuclease motif. AeCRN13 binds DNA, leading to DNA damage and eventual cell death (Ramirez-Garcés et al., 2016). Mutations of key residues in this motif abolish DNA binding and reduce the susceptibility conferred by the effector. Intriguingly, the amphibian fungal pathogen *Batrachochytrium dendrobatidis* contains a homologous effector (BdCRN13), which is thought to function similarly (Ramirez-Garcés et al., 2016). Finally, the *P*. *sojae* effector PsAvr3b has been shown to have nudix hydrolase activity, which is required for its virulence function (Dong et al., 2011). Plant nudix hydrolases can act as negative regulators of immunity, and a recent finding suggests that their enzyme activity benefits pathogen colonization of the host. Indeed, the fungal effector Pst18363 from *Puccinia striiformis* f. sp. *tritici* interacts with and stabilizes the wheat nudix hydrolase TaNUD23, suppressing reactive oxygen species (ROS) accumulation and thereby aiding fungal infection (Yang et al., 2020) (Figure 2).

However, most fungal and oomycete effectors are small proteins with no known functional domains or enzyme activities. Presumably, these effectors are unable to directly modify their targets. We hypothesize that they may instead act as small interfering adaptor proteins that impede or block sites in host proteins that are crucial for enzyme activity or post-translational modifications. They may prevent complex formation, facilitate or disrupt the formation of specific complexes, or alter host protein localization or stability. Does the literature support this?

### Modulation or Utilization of Host Enzyme Activities

One potential effector mode of action is to inhibit or modulate the enzyme activity of host protein targets. Indeed, the RxLR effectors PexRD2 and Pi22926 from *Phytophthora infestans* interact with the kinase domains of StMAP3Kϵ and StMAP3Kβ2, respectively. This inhibits kinase activity and prevents downstream mitogen-activated protein kinase (MAPK) defense signaling (King et al., 2014, Ren et al., 2019). The fungal effector VdSCP41 inhibits the induction of defense genes by calmodulin-binding transcription factors (TFs) CBP60g and SARD1 (Qin et al., 2018). The plant peptidyl-prolyl *cis-trans* isomerase (PPIase) FKBP15-2 is a positive regulator of endoplasmic reticulum (ER) stress-triggered plant immunity. The *Phytophthora capsici* effector Avr3a12 interacts with FKBP15-2 and inhibits its PPIase activity *in vitro* (Fan et al., 2018). By contrast, the interaction of the *Melampsora lini* effector AvrL567-A with the flax cytosolic cytokinin oxidase LuCKX1.1 was demonstrated to increase its catalytic activity against its substrates (Wan et al., 2019). Structural analysis suggests that AvrL567-A binding may alter substrate access to the catalytic site, thereby modifying enzyme activity. Cytokinins are involved in growth and development but have known roles in immunity (Naseem et al., 2015). How AvrL567-A action on LuCKX1.1 activity influences defense is not yet known (Figure 2). Finally, the *P*. *infestans* RxLR effector Pi04314 contains a canonical R/KVxF motif found in regulatory subunits that allows them to attatch to the catalytic subunits of protein phosphatase one (PP1c). Pi04314 utilizes PP1c activity in the host nucleus to suppress transcriptional responses regulated by the hormones JA and SA (Boevink et al., 2016a).

### Altering Host Protein Stability

There are several examples of filamentous phytopathogen effectors influencing the activity of their targets but, in some cases, this seems to occur via regulating the stability of host targets. The 26S proteasome is responsible for the degradation of proteins targeted for ubiquitination by E3 ubiquitin ligases. The effector AvrPiz-t from the fungal pathogen *Magnaporthe oryzae* binds the RING-type E3 ligases APIP6 and APIP10 in rice. This interaction results in the ubiquitination of AvrPiz-t and subsequent 26S proteasome-mediated turnover of the effector/E3 ligase complexes (Park et al., 2012, Park et al., 2016). The oomycete effector HaRxL44 from *Hyaloperonospora arabidopsidis* interacts with the transcriptional Mediator subunit MED19a. MED19a acts as a positive regulator of immunity to *H*. *arabidopsidis*, and the interaction with HaRxL44 results in its proteasome-dependent degradation (Caillaud et al., 2013). More recently, *P*. *sojae* has been shown to suppress ethylene (ET) biosynthesis through the action of the effector PsAvh238 (Figure 2). This effector interacts with type 2 1-aminocyclopropane-1-carboxylic acid synthases and prevents their activity by destabilizing the protein in a 26S proteasome-dependent manner (Yang et al., 2019).

On the other hand, filamentous phytopathogen effectors may also increase the abundance or stability of their host protein targets to facilitate infection. The *P*. *sojae* effector PsAvh262 can suppress ER stress-triggered cell death by stabilizing binding immunoglobulin proteins (BiPs), which play roles in the unfolded protein response (Jing et al., 2016). The *P*. *infestans* effector Pi04089 interacts with and stabilizes its host protein target StKRBP1, a nuclear-localized RNA-binding protein that negatively regulates immunity and promotes *P*. *infestans* colonization (Wang et al., 2015). Similarly, PsAvr3c from *P*. *sojae* stabilizes soybean serine/lysine/arginine-rich proteins (GmSKRPs). These proteins localize to a complex with spliceosome components, and effector action is thought to modify host pre-mRNA splicing (Huang et al., 2017) (Figure 2).

### Disruption of Protein Complexes

A further mode of action of filamentous phytopathogen effectors is to disrupt the formation of biologically active protein complexes to subvert immunity (Figure 2). Good examples of this include the fungal effector jasmonate/ethylene signaling inducer 1 (Jsi1) from *U*. *maydis* (Figure 2). Jsi1 contains an EAR motif (DLNxxP), which binds to co-repressors Topless and Topless-related (TPL/TPR) proteins. This leads to an induction of ET signaling by preventing the formation of the ethylene response factor (ERF)–TPL/TPR complex. Interestingly, several fungal effectors have been predicted to possess EAR motifs, suggesting their potential to also bind TPL/TPR proteins (Darino et al., 2019). Recently, the *P*. *capsici* effector PcAvh103 has been shown to interact with defense regulator enhanced disease susceptibility 1 (EDS1). The binding of PcAvh103 to the EDS1 lipase domain stops EDS1–PAD4 association, thus effectively preventing downstream signaling mediated by this complex (Li et al., 2020). The RxLR effector PexRD54 from *P*. *infestans* hijacks autophagosomes through interaction with autophagy protein ATG8CL, displacing the immune-associated autophagy cargo receptor Joka2 from the complex (Dagdas et al., 2016, Dagdas et al., 2018). As a further example, non-expressor of pathogenesis-related 1 (NPR1) is a master regulator of transcriptional responses in immunity and forms various host protein complexes. The effector PNPi from *Puccinia striiformis* interacts with NPR1 from wheat. PNPi binding to NPR1 competes with the binding of the TF TGA2.2 and leads to lower levels of defense gene induction (Wang et al., 2016a).

### Target Relocalization

In addition to the disruption of complex formation, some effectors function by altering the subcellular localization of their host targets. Again, taking NPR1 as an example, interaction with the *P*. *capsici* effector PcRxLR48 promotes NPR1 nuclear localization and stabilization to disrupt NPR1 function (Li et al., 2019a). Moreover, PsAvh52 relocalizes the soybean transacetylase GmTAP from the cytoplasm to the nucleus (Figure 2), where it then acetylates histones H3 and H2A to promote early *P*. *sojae* colonization (Li et al., 2018). Effectors not only act to promote a particular subcellular localization, they can also prevent the normal host protein localization pattern occurring during defense. The effector PstGSRE1 from *P*. *striiformis* interacts with and inhibits the nuclear localization of the wheat ROS-associated TF TaLOL2 (Qi et al., 2019). Furthermore, effectors from oomycete pathogens *P*. *infestans* and *Bremia lactucae* have been shown to interact with and prevent the nuclear translocation of ER-associated tail-anchored NAC TFs (McLellan et al., 2013, Meisrimler et al., 2019) (Figure 2).

## Effector Targets that Act as Susceptibility Factors

Many pathogen effectors are expected to suppress host targets that positively regulate plant immunity (Deslandes and Rivas, 2012). However, some pathogen effectors target S factors, proteins whose activity in some way promotes infection (Van Schie and Takken, 2014, Boevink et al., 2016b). Historically, NLR-like proteins, which were activated by effectors from necrotrophic pathogens, were described as S factors, as the cell death they trigger benefits the pathogen infection cycle (Wang et al., 2014). For example, the presence of the effector ToxA from *Pyrenophora tritici-repentis* is detected by Tsn1, which is a serine/threonine kinase nucleotidebinding leucine-rich repeat (S/TPK-NBS-LRR) protein (Faris et al., 2010, See et al., 2018). TSN1 is a major S factor involved in ToxA-triggered cell death, which favors necrotrophic pathogen growth (Virdi et al., 2016). In this review, however, we focus on the emerging area of S factors that are targeted by effectors from biotrophic or hemibiotrophic filamentous pathogens. Such targets include S factors that are negative regulators of immunity in the host (Table 1).

**Table 1:** Host Targets that Negatively Regulate Immunity.

Effector	Species	Target	Biological function	Reference
Pi17316	*Phytophthora infestans*	StVIK	Signaling	Murphy et al. (2018)
AvrLm1	*Leptosphaeria maculans*	BnMPK9	Signaling	Ma et al. (2018)
PiAvr2	*Phytophthora infestans*	StBSL1/2/3	Signaling	Saunders et al., 2012, Turnbull et al., 2017, Turnbull et al., 2019)
PvRXLR131	*Plasmopara viticola*	BKI1	Signaling	Lan et al. (2019)
RxLR207	*Phytophthora capsici*	BPA1, BPL	Signaling	Li et al. (2019b)
PsAvh262	*Phytophthora sojae*	GmBIP1/2/3/4	Signaling	Jing et al. (2016)
Pi02860	*Phytophthora infestans*	NRL1	Protein regulation	Yang et al., 2016, He et al., 2018
Pi04314	*Phytophthora infestans*	StPPIc1/2/3	Protein regulation	Boevink et al. (2016a)
PpEC23	*Phakopsora pachyrhizi*	GmSPL12l	Transcription	Qi et al. (2016)
MiSSP7	*Laccaria bicolor*	JAZ6	Transcription	Plett et al. (2014)
PsAvh52	*Phytophthora sojae*	GmTAP1	Transcription	Li et al. (2018)
Pi04089	*Phytophthora infestans*	StKRBP1	RNA trafficking/processing	Wang et al. (2015)
PsAvr3c	*Phytophthora sojae*	GmSKRP1/2	RNA trafficking/processing	Huang et al. (2017)
Avr1-CO39, Avr-Pia, Avr-PikD	*Magnaporthe oryzae*	Pi21	Cellular trafficking	Fukuoka et al., 2009, Ortiz et al., 2017, Guo et al., 2018
ROPIP1	*Blumeria graminis* f. sp. *hordei*	HvRACB	Cellular trafficking	Nottensteiner et al. (2018)
PsAvr3b	*Phytophthora sojae*	GmCYP1	Metabolism	Dong et al., 2011, Kong et al., 2015
Pst18363	*Puccinia striiformis* f. sp. *tritici*	TaNUDX23	Metabolism	Yang et al. (2020)
PiAvr3a, PsAvr1b, PcAvr3a1, PcAvr3a12	*Phytophthora infestans*, *sojae* and *capsici*	AtCAD7	Metabolism	Li et al. (2019c)

**Figure 3:**
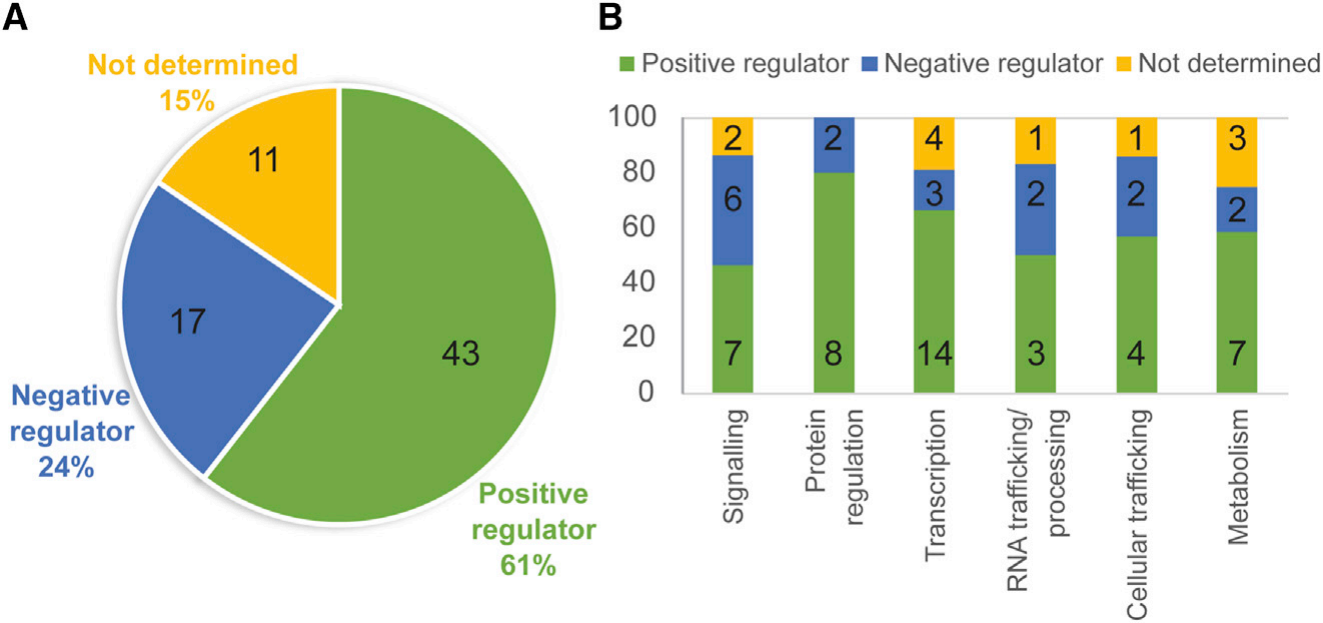
Effectors Target Both Positive and Negative Regulators of Immunity. **(A)** The pie chart shows the percentage of effectors that interact with host proteins that positively or negatively regulate immunity; actual effector numbers are indicated on the chart. **(B)** The stacked column bar chart shows the percentage of effectors that target both positive and negative defense regulators from each biological category; actual effector numbers are indicated on the chart.

Of the filamentous phytopathogen effectors shown in Supplemental Table 1, 61% target positive immune regulators, 24% target negative immune regulators, and the function of the remaining 15% of effector targets is yet to be determined (Figure 3A). Interestingly, both positive and negative immune-regulating host targets belong to each of the biological categories targeted by effectors from fungal and oomycete pathogens (Figure 3B), perhaps indicating the need to enhance or suppress different aspects of these processes.

### S Factors that Negatively Regulate Plant Immunity

Some S factors targeted by effectors encode endogenous negative regulators of plant immunity (Table 1). It is perhaps not surprising that pathogens have evolved effectors to exploit these proteins, as they will be fine-tuned to efficiently suppress defenses when the host no longer needs them. Several examples exist in which independent expression of either effectors from *P*. *infestans* or their targets can suppress cell death triggered by the MAMP INF1. For example, both the effector Pi17316 and its host target, the MAP3K StVIK, suppress INF1-triggered cell death (ICD). Critically, if *StVIK* is silenced, the ability of Pi17316 to suppress ICD is compromised, showing that this effector activity depends on its target (Murphy et al., 2018). In addition, PiAVR2 from *P*. *infestans* also suppresses ICD. It interacts with three BRI1-suppressor 1-like (BSL) family proteins in potato, which are phosphatases that regulate brassinosteroid signaling. BSL1 and BSL3 suppress ICD and promote *P*. *infestans* virulence via their induction of the transcription factor CHL1. The knockdown of both *BSL1* and *BSL3*, or *CHL1* alone, compromises ICD suppression by PiAVR2. PiAVR2 thus exploits the crosstalk and mutual antagonism between growth and development on one hand, and immunity on the other, that is regulated by the BSLs (Turnbull et al., 2017, Turnbull et al., 2019). As a further example, the transient expression of either the effector Pi02860 or its target NRL1 leads to ICD supression, and again the capacity for the effector to do this depends on the target (Yang et al., 2016). Recently, it has been shown that NRL1, a ubiquitin E3 ligase predicted to be activated by blue light phototropin receptors, promotes the ubiquitination and proteasome-mediated turnover of the guanine exchange factor SWAP70, which is a positive immune regulator required for ICD (He et al., 2018).

In addition to the increasing number of effectors from *P*. *infestans* that target S factors, this strategy is evident for *P. sojae* also. As indicated above, the spliceosome-associated GmSKRPs from soybean are negative regulators of immunity involved in changing pre-mRNA splicing and are targeted by the effector PsAvr3c from *P*. *sojae* (Huang et al., 2017) (Figure 2). The *P*. *sojae* effector PsAvh52 promotes the relocalization of soybean GmTAP to the nucleus to facilitate histone acetylation, leading to epigenetic changes that suppress immunity (Figure 2) (Li et al., 2018). Furthermore, PsAvh262 targets GmBiP1/2/3/4 proteins that are suppressors of ER stress-associated cell death, leading to enhanced pathogen colonization (Jing et al., 2016).

Effectors from biotrophic and hemibiotrophic fungi also target S factors that suppress immunity. Effector candidate PpEC23 from the soybean rust fungus *Phakopsora pachyrhizi* interacts with the SQUAMOSA promoter-binding-like protein 12-like (GmSPL12l). GmSPL12l is a negative regulator of immunity that Qi et al. (2016) propose is utilized by PpEC23 during infection. In addition, as mentioned above, the stripe rust effector Pst18363 targets and stabilizes TaNUDX23, which acts as a negative regulator of immunity, to promote disease (Yang et al., 2020). As a further example, the *Leptosphaeria maculans* effector AvrLm1 targets the MAPK BnMPK9, promoting its phosphorylation. BnMPK9 is described as a negative regulator of immunity that suppresses SA-mediated defenses, and this activity is supported by AvrLm1 interaction (Ma et al., 2018). Finally, Pi21 in rice is proposed to be targeted by several avirulence effectors from *M. oryzae* (Ortiz et al., 2017, Guo et al., 2018). Pi21 is a putative heavy metal-binding domain-containing protein that negatively regulates immunity (Fukuoka et al., 2009). Intriguingly, the effector-binding regions of Pi21 have been integrated as decoys into corresponding R proteins so that interaction with AVR-Pia, AVR-C039, and AVR-PikD triggers ETI (Ortiz et al., 2017, Guo et al., 2018).

### S Factors Facilitate Pathogen Penetration

To infect plants, a pathogen needs to breach the host defensive barriers, such as plant cell walls. Some fungi and oomycetes penetrate the leaf surface using specialized infection structures, such as appressoria, to initiate infection. Many also develop haustoria, which facilitate molecular exchange between the pathogen and its host. Some effectors target S factors to facilitate penetration, and this may define whether a plant is a suitable host. For example, the powdery mildew effector ROPIP1 was shown to target the barley S factor ROP GTPase RACB, which supports fungal penetration by provoking host cell microtubule disorganization (Nottensteiner et al., 2018).

## Common Targets of Effectors

Plants have conserved proteins at the center of signaling and regulatory networks. These proteins are highly connected to other plant proteins and are predicted to influence their functions through physical interactions. These “regulatory hub” proteins have important roles in the control of normal growth and development, responses to biotic and abiotic stress, or the crosstalk between them. A key study defined the effector interactome of type III effectors (T3Es) from *Pseudomonas syringae* and RxLR effectors from the oomycete *H*. *arabidopsidis* with the candidate proteins they target in Arabidopsis (Mukhtar et al., 2011). This study, and a subsequent one that introduced candidate effectors from the ascomycete fungus *Golovinomyces orontii* (Weßling et al., 2014), used a matrix yeast-2-hybrid system to investigate the protein–protein interactions between thousands of Arabidopsis proteins and effectors from these three kingdoms of pathogen. These studies revealed a core set of nine regulatory hub plant proteins that are potentially targeted by effector proteins from bacterial, fungal, and oomycete pathogens, and 24 host proteins that interacted with effectors from any two of the pathogens, suggesting effector convergence onto key targets to promote microbial pathogenic fitness. Among Arabidopsis proteins that interacted with effectors from all three pathogens were response to low sulfur LSU2, anaphase-promoting complex 8 (ACP8), the JA regulator JAZ3, the CSN5a subunit of the COP9 signalosome, and the TCP family TFs TCP13, TCP14, and TCP15 (Weßling et al., 2014). TCP14 interacted with a remarkable 60 candidate effectors. Although these interactions were not verified in this study, TCP14 has been shown to interact with the *Phytophthora capsici* CRN12-997 effector in tomato and the *P. syringae* effector HopBB1 in Arabidopsis (Stam et al., 2013, Yang et al., 2017). Interestingly, knocking out both *TCP13* and *TCP14* in Arabidopsis led to enhanced disease susceptibility (EDS) phenotypes with *H*. *arabidopsidis* and *G*. *orontii* but an enhanced disease resistance (EDR) phenotype with *P*. *syringae* (Weßling et al., 2014). This observation suggests that the TCP13 and TCP14 proteins could act as positive regulators of immunity to the filamentous pathogens but as negative regulators to a bacterial pathogen. Several knockout mutants of other targets of multiple effectors from the different pathogens yielded contrasting EDR and EDS phenotypes (Weßling et al., 2014) similar to those observed with TCP13 and TCP14, perhaps indicating that there are different requirements for infection by these distantly related pathogens.

Among immune-associated regulatory hubs are kinases BAK1 and BIK1, which control multiple PRRs for MAMP sensing and signaling (Heese et al., 2007, Lu et al., 2010, Roux et al., 2011) and also regulate brassinosteroid sensing that leads to growth and development (He et al., 2013, Lin et al., 2013). The *P*. *syringae* effectors AvrPto, AvrPtoB, HopF2, and HopB1, target BAK1 in Arabidopsis (Shan et al., 2008, Zhou et al., 2014, Li et al., 2016), and the *Xanthomonas oryzae* effector Xoo2875 targets OsBAK1 in rice (Yamaguchi et al., 2013). The *P*. *syringae* effector AvrPphB (Zhang et al., 2010) and the *Xanthomonas campestris* effector AvrAC (Feng and Zhou, 2012) target BIK1. The widely conserved effector NIS1, found in fungi such as *Colletotrichum tofieldiae* and *M. oryzae*, can target both BAK1 and BIK1, inhibiting their kinase activities and thereby impairing PTI signaling (Irieda et al., 2019). As another example, the immune regulator SGT1, which is conserved across diverse plant lineages, is targeted by bacterial phytopathogen effectors, such as *P*. *syringae* AvrB (Cui et al., 2010) and the fungal effector See1 from *U*. *maydis*, preventing its MAPK-triggered phosphorylation (Redkar et al., 2015). In addition, the core SA regulator NPR1 is not only targeted by the fungal effector PNPi (Wang et al., 2016a) and the oomycete effector PcRxLR48 (Li et al., 2019a) but also by the bacterial effector AvrPtoB from *P. syringae* (Chen et al., 2017). Finally, the transcriptional repressor Topless (TPL), which interacted with the oomycete effector HaRXLR21 as that has been reported by Weßling et al. (2014), is a target of the fungal effectors Jsi1 (Darino et al., 2019) and MLP124017 (Petre et al., 2015). There is thus increasingly compelling evidence that effectors from pathogenic microbes of different kingdoms have converged onto conserved, key regulators of immunity.

## Multifunctional Effectors

**Figure 4:**
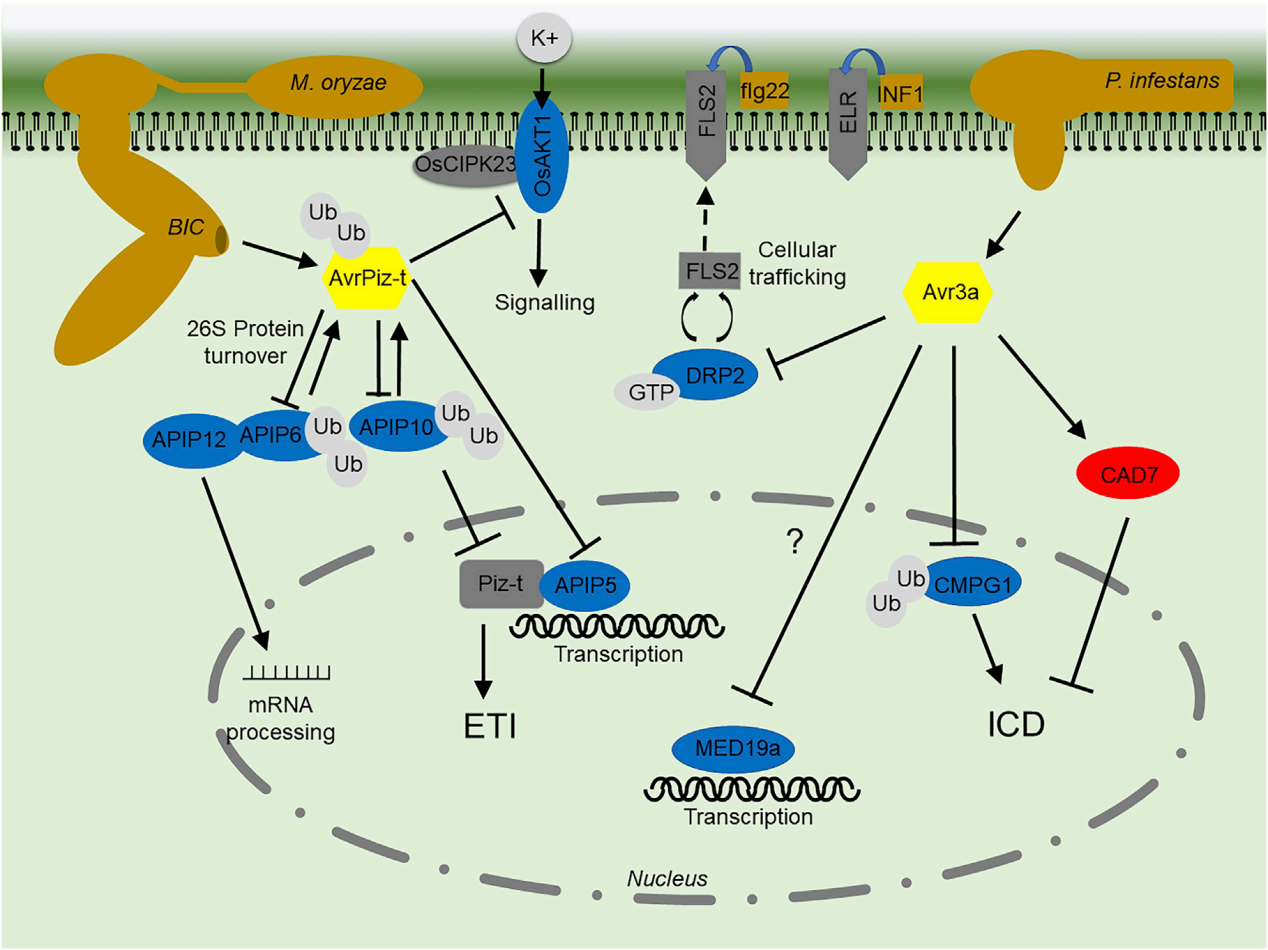
Effectors that Interact with Multiple Host Targets and Interfere with Different Processes. Effectors are shown in yellow. Effector targets that are positive regulators of immunity are shown in blue, and negative regulators of immunity are shown in red. Pathogens and MAMPs are shown in brown. BIC, biotrophic interfacial complex; Ub, ubiquitin; K^+^, potassium ions; ICD, INF1-triggered cell death.

We have shown that pathogens use numerous effectors to modify various aspects of plant immune systems, including plant cell signaling, transcription, protein processing and turnover, RNA trafficking and processing, cellular trafficking, and metabolism. Various bacterial effectors target and modify multiple host proteins via interactions with the different domains that they contain (Deslandes and Rivas, 2012, Lee et al., 2013, Büttner, 2016;Khan et al., 2018). Recent work has found that individual filamentous phytopathogen effectors can also target different host proteins, apparently interfering with distinct cellular processes to suppress plant immunity. Examples of these multifunctional effectors and their targets are shown in Figure 4.

The RxLR effector AVR3a from *P*. *infestans* can suppress ICD through its interaction with the E3 ligase CMPG1 (Bos et al., 2010, Gilroy et al., 2011). AVR3a also associates with GTPase dynamin-related protein 2 (DRP2). This results in the suppression of PTI triggered by flg22 perception by inhibiting the endocytosis of the active FLS2 receptor (Chaparro-Garcia et al., 2015). Recently, Li et al. (2019c) found that the plant cinnamyl alcohol dehydrogenase 7 (CAD7)-like subfamily members are S factors that are the common targets of several Avr3a-like effectors from different *Phytophthora* species in different hosts. These effectors stabilize AtCAD7 and attenuate PTI responses, including ROS generation, callose deposition, and marker gene expression (Li et al., 2019c). Taken together, these observations suggest that Avr3a suppresses PTI by three different mechanisms. Intriguingly, one of the original Avr3a-interacting potato proteins from Bos et al. (2010) is a homolog of the mediator subunit MED19a, which is a verified target of the *H*. *arabidopsidis* effector HaRxL44 (Caillaud et al., 2013), raising yet another potential function for this effector.

In addition to interacting with two E3 ligases APIP6 and APIP10 (Park et al., 2012, Park et al., 2016), the effector AvrPiz-t from the rice blast fungus *M. oryzae* directly interacts with three unrelated proteins (Figure 4). AvrPiz-t suppresses the transcriptional activity of the bZIP-type TF APIP5 and decreases its protein accumulation (Wang et al., 2016b). AvrPiz-t also interacts with the nucleoporin-like protein APIP12 required for *PR*-gene transcript accumulation (Tang et al., 2017). Recently, AvrPiz-t was found to interact with the rice K^+^-channel protein OsAKT1 and suppresses OsAKT1-mediated K^+^ fluxes (Shi et al., 2018).

Throughout the interdependent evolutionary history of plants and pathogens, plants have made use of a tiered immune system to protect against a wide range of microbial life forms to which they are exposed. Therefore, it is not surprising that some effectors have evolved to be multifunctional to suppress plant immunit in different ways. One thing these multifunctional effectors have in common is that they can be recognized by the host’s R proteins. It is tempting to speculate that by interfering with many different processes, effectors enhance the risk that they may trigger R protein surveillance systems.

## Do Effectors from Filamentous Symbionts/Mutualists and Pathogens Target Similar Processes?

An emerging area of interest in plant–microbe interactions concerns how symbionts/mutualists form a molecular relationship with their hosts during colonization. These organisms can improve nutrient or water availability to their host plants in exchange for food and yet are equipped with the same MAMPs and will therefore trigger immunity. Many symbiotic associations are as intimate as those for pathogens and involve long-term colonization of the host, yet there is rarely massive elicitation of plant immunity leading to host resistance.

Recent evidence shows that symbiotic fungi also possess effector proteins to manipulate the host immune system. For example, arbuscular mycorrhizal fungi (AMF) form the most widespread mutualistic symbioses with plant roots. Bioinformatics studies found overlapping effector candidates for AMF *Rhizophagus irregularis* (formerly *Glomus intraradices*) and *Rhizophagus clarus* (Toro and Brachmann, 2016). RiSLM is an apoplastic LysM-type effector that interferes with chitin-triggered immunity in a manner similar to the LysM effectors from many fungal pathogens (Zeng et al., 2020). SP7, an intracellular effector secreted by *R*. *irregularis*, interacts with ERF19 in the nucleus. This leads to a reduction in the induction of ERF19-mediated defense genes, which benefits mycorrhizal colonization (Kloppholz et al., 2011). Similarly, *R*. *proliferus* effectors RP8598 and RP23081 can interact with ERF19 from five plant species (Prasad Singh et al., 2019). Another study has reported that ERFs can also be targeted by the *Xanthomonas* type III effector XopD (Kim et al., 2013). Therefore, it is evident that in long-term biotrophic relationships with hosts, AMF also needs to suppress plant defense responses.

Ectomycorrhizal (ECM) fungi form mutualistic symbioses with many tree species. Mycorrhiza-induced small secreted protein 7 (MiSSP7), which is encoded by *Laccaria bicolor*, interacts with the *Populus* jasmonate (JA) Zim-domain 6 (PtJAZ6) protein. PtJAZ6 is a negative regulator of JA induced transcription, and interaction with MiSSP7 inhibits its JA-triggered degradation, thus reducing JA signaling (Plett et al., 2014). As the immune-suppressing activity of PtJAZ6 is maintained by the action of MiSSP7, it can be regarded as an S factor (Table 1). JAZ repressors are also targeted by pathogen effectors from the bacterium *P*. *syringae*, the oomycete *H*. *arabidopsidis*, and the fungus *G*. *orontii* (He et al., 2004, Jiang et al., 2013, Gimenez-Ibanez et al., 2014, Weßling et al., 2014). Although we still know relatively little about filamentous symbionts/mutualists, it is apparent that they possess effectors that target processes similar to those targeted by pathogens to suppress plant immunity.

## Concluding Remarks and Future Perspectives

### The Biochemical Activities of Effectors

Intracellular fungal and oomycete effectors are generally small proteins that alter the fate of host target proteins, changing their stability or activity, where they localize in the host cell, and the complexes that they form with other proteins. Whereas some effectors may inhibit target activity, others may enhance or utilize that activity. More detailed analyses are needed to understand the structural relationships between effectors and their targets, as well as the precise biochemical consequences of effector interactions upon host protein targets. A key question to address is: how many of these fungal and oomycete effectors prevent or promote post-translational modifications that regulate target stability, activity, location, and complex formation?

### The Immune-Regulating Network in the Host

The targets of filamentous pathogen effectors represent many diverse biological and biochemical processes. Nevertheless, in many cases, the exact functions of these targets and how that is related to immunity are unknown. There needs to be a major shift from identifying the targets of effectors to more clearly determining the roles and functions of these host proteins. What is even less clear is how the activities of effector targets relate to each other. To what extent is there a network of immune regulation, as suggested in the large-scale target identifications of Mukhtar et al. (2011) and Weßling et al. (2014)? How many effectors, working in concert, are needed to perturb the regulatory “flux” passing through the network to activate the many diverse immune outputs? What combinations of effectors, and therefore what combinations of key pressure points in the immune network, must be altered to create a susceptible environment? Are apparent multifunctional effectors with multiple host protein interactors targeting distinct processes in the overall network, or are some of these targets related in their activity, perhaps even parts of the same protein complex?

### S Factors, Common Targets, and Different Colonization Strategies

It is increasingly apparent that not all targets of fungal and oomycete effectors are positive regulators of immunity. Some are so-called S factors, including some host proteins that negatively regulate immunity. To what extent are these host proteins that regulate the crosstalk between processes involved in growth and development on the one hand, and stress responses on the other? Are some of these targets the regulatory switches of the resource allocation among these higher-level processes within the plant? A deeper understanding of S factors can help develop new strategies to control disease, for example, by removing a protein that the pathogen needs for susceptibility, as opposed to adding in a factor involved in recognition and resistance activation, such as an R protein.

The increased efforts to identify targets of effectors from filamentous pathogens have revealed several host proteins that are commonly targeted by different species, genera, and kingdoms of microbial life. Although, some common targets are hubs that positively regulate immunity, such as BAK1, others like TCP14, potentially regulate a number of processes. In such cases, it is not yet clear whether effectors from very distantly related pathogens manipulate these targets in similar or in highly contrasting ways. As indicated above, TCP14 knockout lines have EDS phenotypes with *H*. *arabidopsidis* and *G*. *orontii* and an EDR phenotype with *P. syringae* (Weßling et al., 2014), emphasizing the potential for (hemi-)biotrophic pathogens to have different infection requirements. This is potentially also the case for necrotrophs versus biotrophs, with S factors targeted by the former being positive immune regulators targeted by the latter, and vice versa. Future studies should investigate the different strategies used by effectors from biotrophic and necrotrophic pathogens, as well as from symbionts, to manipulate biological processes in the host for successful colonization.

### Differences in Host Range

It is also unclear why some oomycete and fungal pathogens with similar infection strategies have broad or narrow host ranges. Whether this is due to differences in the ways that effector targets are guarded by R proteins in different plant species or whether targets are evolving to evade effector manipulation remain to be explored. In addition, the roles of effectors and their targets in determining host range and non-host resistance are not clear. It is increasingly pressing to understand how emerging pathogens, such as the promiscuous tree pathogen *Phytophthora ramorum*, can infect hosts with which they have presumably not co-evolved. Understanding of the molecular mechanisms underlying host range and host-jump potential will benefit from the increasing efforts to identify and characterize the targets of filamentous pathogen effectors.

## Funding

We are thankful for financial support from the 10.13039/501100000268Biotechnology and Biological Sciences Research Council grants BB/P020569/1, BB/N009967/1, and BB/L026880/1, the 10.13039/501100000781ERC-Advanced grant PathEVome (787764), and the Scottish Government 10.13039/100011310Rural and Environment Science and Analytical Services Division. Q.H. is grateful for the Project 2662020YLQD001 supported by the Fundamental Research Funds for the Central Universities and Research Start Fund 105/11042010004 for High-Level talents in Huazhong Agricultural University, China.

## Supplemental Information

Supplemental Table 1. Filamentous Phytopathogen Intracellular Effectors and Their Host Targets
